# Structure-Function Correlation of Deep-Learning Quantified Ellipsoid Zone and Retinal Pigment Epithelium Loss and Microperimetry in Geographic Atrophy

**DOI:** 10.1167/iovs.66.3.26

**Published:** 2025-03-11

**Authors:** Klaudia Birner, Gregor S. Reiter, Irene Steiner, Azin Zarghami, Amir Sadeghipour, Simon Schürer-Waldheim, Markus Gumpinger, Hrvoje Bogunović, Ursula Schmidt-Erfurth

**Affiliations:** 1 Department of Ophthalmology and Optometry, Medical University of Vienna, Vienna, Austria; 2 Center for Medical Data Science, Institute of Medical Statistics, Medical University of Vienna, Vienna, Austria; 3 RetInSight, Vienna, Austria

**Keywords:** artificial intelligence, photoreceptors, optical coherence tomography (OCT), functional imaging, retina

## Abstract

**Purpose:**

The purpose of this study was to define structure-function correlation of geographic atrophy (GA) on optical coherence tomography (OCT) and functional testing on microperimetry (MP) based on deep-learning (DL)-quantified spectral-domain OCT (SD-OCT) biomarkers.

**Methods:**

Patients with GA were prospectively examined by SD-OCT (Spectralis, 97 B-scans) and two microperimetry devices (MP3 and MAIA) in two combined test runs each. DL-algorithms measured the ellipsoid-zone thickness (EZT), ellipsoid-zone loss (EZL), hyper-reflective-foci (HRF) volume, drusen-volume (DV), and retinal-pigment-epithelium loss (RPEL) area. Pointwise co-registration was established between all stimuli and the location on OCT. A multivariable mixed-effect model with variable selection was used to identify pointwise retinal sensitivity (PWS) changes for each biomarker, accounting for age and eccentricity.

**Results:**

Three thousand six hundred stimuli points were collected and correlated with 1940 OCT B-scans in 20 eyes of 20 patients. PWS was significantly lower in stimuli with EZL without RPEL (−2.81, 95% confidence interval [CI] = −3.72 to −1.91 decibel [dB], 0 degrees, *P* < 0.0001) and in areas with both EZL and RPEL (−10.03, 95% CI = −10.96 to −9.11 dB, 0 degrees, *P* < 0.0001) compared to areas without any atrophy. Increased EZT had a significant positive effect on PWS (0.34, 95% CI = 0.32 to 0.36 dB/µm, *P* < 0.0001). Structure-function correlations were consistent throughout all levels of eccentricity with *P* < 0.001. Drusen and HRF volume, but not age, were associated with reduced PWS.

**Conclusions:**

Functional impairment by MP was associated with defined morphological changes as quantified by DL on OCT. PR degeneration seen as EZL alone impairs the function on MP examinations. The combination of DL-based SD-OCT biomarker assessment and MP appear suited for evaluation of retinal function beyond visual acuity for disease monitoring.

Regulatory approval of complement inhibition as the first treatment for non-exudative age-related macular degeneration (AMD) introduces an urgent need for optimal morphological and functional monitoring and progression assessment beyond conventional visual acuity testing.^1^^–^^3^ Optical coherence tomography (OCT) allows for a high-resolution noninvasive display of retinal morphology for precise detection and monitoring of AMD.^4^ International OCT-based consensus for geographic atrophy (GA)-specific biomarkers was established in recent years highlighting retinal pigment (RPE) attenuation and photoreceptor (PR) degeneration as hallmark OCT-related signs of disease activity.^5^ Results from interventional clinical trials in GA show a strong relation between disease progression and structural OCT changes in the GA lesion area. In 1988, Sarks et al. demonstrated that PR damage exceeds retinal pigment epithelium loss (RPEL) in a case series,^6^ which was confirmed in more recent histological studies.^7^ Previously described PR attenuation in perilesional areas of GA is of high interest for understanding disease morphology and therapeutic response.^8^^,^^9^ Most recently, advances in deep-learning (DL) models additionally provide objective and fast quantification of changes in the perilesional area at the level of the ellipsoid zone, namely ellipsoid zone loss (EZL) surrounding the RPEL area as pathognomonic OCT biomarkers in GA.^10^^–^^12^ The primary location of the often parafoveal onset in GA leads to a lack of detection of progressive functional impairment by the widely used (best-corrected) visual acuity (VA) testing derived from the fovea. Vice-versa, in case of a complete foveal wipeout, VA remains unchanged even if significant disease progression occurs or is prevented. This discrepancy raises the need to establish better-suited functional assessment methods in GA, not only for regulatory approval of novel therapeutic targets but also for clinical patient monitoring.^3^ In recent literature, microperimetry (MP) is the most commonly studied test for objective functional GA assessment. However, defining adequate metrics correlated to GA progression remains a challenge.^13^ Commonly described approaches include mean retinal sensitivity, overall assessment of scotomatous points, and high-density grids focused on various macular areas of interest.^14^^,^^15^ We aim to establish a direct correspondence between focal pointwise retinal sensitivity (PWS) and automatically quantified areas of EZL and RPEL, as well as high-risk GA biomarkers, in two commonly studied MP devices to propose a novel method for personalized functional testing of GA.

## Methods

### Study Population

Patients above 50 years of age were recruited at the Department of Ophthalmology and Optometry at the Medical University of Vienna, Austria. Informed written consent was received prior to inclusion with compliance to the tenets of the Declaration of Helsinki and approval by an independent institutional review board (Ethikkommission, Medizinische Universität Wien, Österreich, Nr. 1399/2021). Patients with both extrafoveal and subfoveal GA as defined on OCT imaging were included. Eligibility was assessed on OCT imaging based on the atrophy definition based on the CAM consortium with complete RPE and outer retinal atrophy (cRORA) defined as an RPE attenuation > 250 µm, choroidal hypertransmission > 250 µm, and overlying photoreceptor degeneration without the evidence of an RPE tear.^5^ The size of GA was defined as the area of RPEL derived from OCT imaging. Sufficient OCT image quality and patient compliance were required for study inclusion. Patients with macular neovascularization, which was defined as no history of anti-VEGF injections and no active exudation on OCT were excluded.^16^ Patients with any ocular comorbidities including any form of glaucoma with a cup-to-disc ratio more than 0.8 or an intraocular eye pressure > 25 millimeters of mercury (mm Hg), media opacity or advanced cataract, in the opinion of the investigator, that could falsify the results and refractive error > −6.0 diopter (D) and > +6.0 D (spherical equivalent) were not eligible for study inclusion. Phakic and pseudophakic patients were included. One eye per patient was included. In cases where both eyes were eligible, the one with the better OCT imaging quality was selected.

### Imaging and Microperimetry Testing

The cohort was imaged using a Spectralis device (Heidelberg Engineering, Heidelberg, Germany) with 97 B-scans in a 20 × 20 degrees macular cube. Patients underwent 4 microperimetry tests during the same visit after pupillary dilation with 0.5% Tropicamid. Two consecutive test runs were conducted using the MP-3 (NIDEK Co., Ltd., Gamagori, Japan) in the photopic standard setting (31.4 asb, 10 cd/m2) and two test runs on the Macular Integrity Assessment device (MAIA; CenterVue S.p.A. [iCare], Padova, Italy) in the mesopic standard setting (4 asb, 1.27 cd/m2) to mirror the clinical standard settings of both MP devices. Fixation stability and intra- and interdevice repeatability of this cohort were previously described by Coulibaly et al.^17^ A 4-2 staircase stimulation strategy was selected, whereas the first stimulus was set at 17 decibels (dB) in both devices and test runs with the device-specific follow-up function were performed in the second run. Sensitivity was assessed in a range from 0 to 34 dB in MP-3 and from 0 to 36 dB in MAIA, respectively. An in-house developed high-density grid consisting of 45 stimuli points centered at the fovea with a stimuli size of Goldmann III for the duration of 200 ms was applied in both settings. The −1 dB values provided by the MAIA device in cases where the patient was unable to recognize the light stimulus were analyzed as 0 dB in the statistical analysis for consistency with the MP-3 device-based lower scale bound.

### Image Analysis

**Figure 1.**
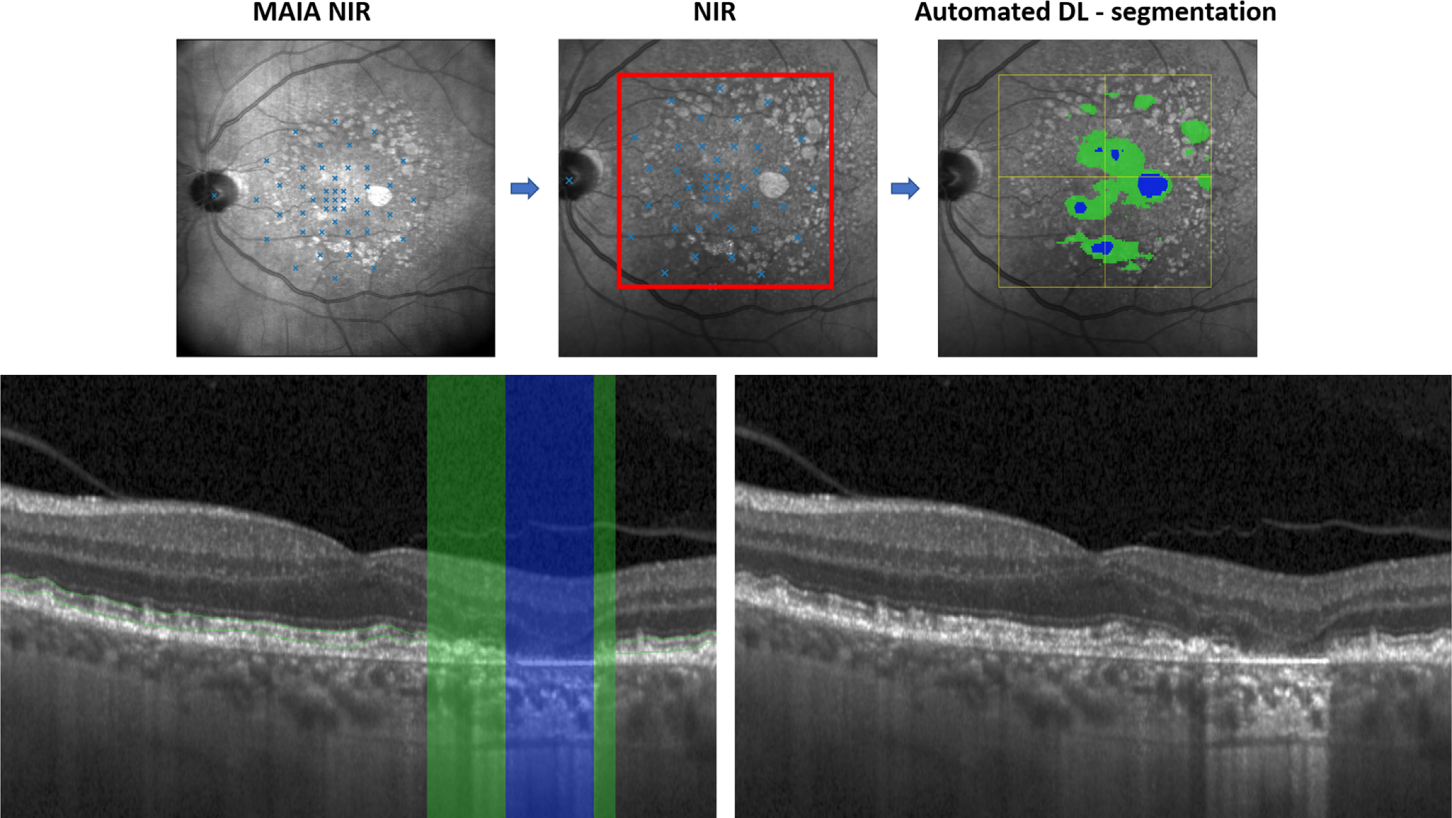
**Registration process** **.** The upper row represents the customized high-density MP grid in MAIA-NIR (*upper row left*) with the registration process from MAIA-NIR to Spectralis-NIR (*left and middle upper rows*). The DL-quantified EZL area is marked in *green*, whereas the area with both EZL and RPEL is marked in *blue* on the Spectralis NIR (*upper row right*). The lower row displays the corresponding OCT B-scan with automated DL-based segmentation of EZL marked in *green* and RPEL marked in *blue* (*lower row left*). The EZ layer thickness was measured between the two *green lines* (*lower row left*) and was defined between the inner boundary ellipsoid zone and the outer boundary interdigitation zone. EZ = ellipsoid zone; EZL = ellipsoid zone loss; RPEL = retinal pigment epithelium loss; NIR = near-infrared reflectance.

Total EZL, defined as loss of the layer between the inner border of the ellipsoid zone and the outer boundary of the interdigitation zone, and RPEL area were segmented in a fully automated manner (Fig. 1) based on previously evaluated and published DL algorithms validated according to the Medical Device Regulations (MDR; GA Monitor, version 1.1.0 RetInSight, Vienna, Austria). Hyper-reflective foci (HRF) and drusen volumes were calculated from automated segmentations of previously published DL-algorithms.^18^^–^^20^ Drusen volume was calculated between Bruch's membrane to the outer border of the RPE^20^ and drusen were graded for refractile and cuticular drusen in each OCT volume.^21^^,^^22^ For the HRF volume, a previously published threshold of 0.06 nL was used to minimize potential false positive segmentation error.^20^

### Pointwise Structure-Function Correlation

The pointwise spatial relation between MP and OCT volumes was established, as previously described in detail.^23^^,^^24^ In short, the MP-3 color fundus photography (CFP) and MAIA near-infrared reflectance (NIR) images are registered with the Spectralis NIR (see Fig. 1) by a semi-automatic approach which is based on the recognition of prominent vascular structures between both images.^25^ The NIR OCT is already registered with the corresponding OCT B-scan, as provided by the Spectralis software. OCT biomarkers were quantified within a 70 µm radius around each respective stimulus point.

### Statistical Analysis

Descriptive statistics were calculated based on mean, standard deviation, and the median, minimum, and maximum for overall biomarkers quantification in all OCT volumes and for pointwise biomarker quantification at the respective 3600 MP stimuli points. The effect of independent metric variables, such as age, eccentricity, EZL area, RPEL area, HRF, and drusen volume, on retinal sensitivity was first calculated in a univariate mixed model. Then, a multivariable model was established using variable selection (R-function dredge, R-package MUMIn_1.47.5).^26^ The global model was a mixed model (R-function lme, R-package nlme^27^) fitted by maximum likelihood (ML) with the patient as the random factor to account for multiple measurements per eye. The independent variables were age, eccentricity, area with EZL and/or RPEL loss (nominal, 3 categories), HRF presence, drusen volume, device, examination run, as well as the interaction between eccentricity and EZL and/or RPEL loss. EZ thickness was not considered in the mixed model due to the high correlation between the EZ thickness and the EZL area.

For model selection, the Bayesian information criterion (BIC) was used. Due to missing values with PWS located outside the OCT scan, 3557 observations out of 3600 observations were included in the multivariable model. Independent variables that were selected in the model with the smallest BIC were included in a mixed model with the patient as a random factor using REML estimation. Estimates with 95% confidence limits and the *P* values of the final model are reported. The correlation between independent variables was analyzed by the Spearman correlation coefficient (rs). The performance of the exploratory model was computed with the marginal coefficient of determination for generalized mixed-effect models *R*^2^_GLMM_, which represents the variance explained by the fixed effects calculated with R-package MuMIn, R-function r.squaredGLMM.^28^ Additionally, a subset analysis was performed for both devices separately without the effect of the device included in the model.

Statistical analyses were carried out with R version 4.3.2.^29^ The significance level was set to alpha = 0.05. It is noteworthy that the analyses and hence the interpretation of the *P* values are exploratory.

## Results

There were 3600 stimuli locations that were collected and correlated with 1940 OCT B-scans from 20 eyes of 20 patients with GA. The mean EZL area was 4.87 ± 3.35 mm² (minimum = 1.49 and maximum = 13.09 mm²) and the mean RPEL area was 2.30 ± 1.86 mm² (minimum = 0.46 and maximum = 7.12 mm²) over the 20 analyzed OCT volumes. Median HRF volume was 24.21 nL (minimum = 14.13 and maximum = 57.85 nL) and median drusen volume was 141.28 nL (minimum = 27.22 nL and maximum = 465.55 nL). Sixteen of 20 patients had refractile drusen and 16 of 20 patients presented with cuticular drusen, which did not concern the same OCT volumes. Seventy percent (14/20) of eyes had foveal sparring. The mean patient age was 77 ± 6 years with 15 female patients and 5 male patients. Eight patients were phakic and 12 patients were pseudophakic. Table 1 provides descriptive statistics for OCT-based biomarkers quantified around MP stimuli and the number of MP stimuli within atrophy area, defined as RPEL and within solely EZL areas. For pointwise measurements, 16% (557/3557) of stimuli points demonstrated EZL without RPEL, 15% (548/3557) showed combined RPEL and EZL, 9% (312/3.557) had an HRF volume > 0.06 nL and 58% (2.090/3557) drusen volume > 0. EZ thickness > 0 was measured in 90% (3209/3557) of stimuli locations. Absolute scotomas of 0 dB were measured in 5% (191/3.557) and scotomas below 10 dB in 11% (402/3557) of stimuli locations. Supplementary Table S1 shows the distribution of stimuli and biomarkers in the central 1 mm Early Treatment Diabetic Retinopathy Study (ETDRS) ring, central 3 mm ETDRS ring, and central 6 mm ETDRS ring.

**Table 1.** Pointwise Descriptive Statistics

	MP-3 Run 1	MP-3 Run 2	MAIA Run 1	MAIA Run 2	Observations
Sensitivity, dB	21.41 ± 7.2	21.80 ± 7.5	18.05 ± 6.87	18.36 ± 6.91	3600
EZ thickness, µm	20.70 ± 11.26	20.92 ± 11.32	20.51 ± 11.30	20.39 ± 11.42	3557
EZL presence	277/898	274/895	274/882	280/882	1105
	31%	31%	31%	31%	
RPEL presence	138/898	132/895	138/882	140/882	548
	15%	15%	16%	16%	
Drusen, nL	0.064	0.077	0.087	0.08	2090
	[6e-17-3.09]	[2e-5-3.16]	[6e-17-2.53]	[3e-17-2.59]	
HRF, nL	0.11	0.10	0.10	0.11	312
	[0.06 – 0.60]	[0.06 – 0.55]	[0.06 – 0.54]	[0.06 – 0.55]	

OCT, optical coherence tomography; PWS, pointwise sensitivity.

Descriptive statistics of biomarkers at the respective PWS points quantified within a 70 µm radius on OCT. Normally distributed data are reported as mean ± standard deviation (SD) and skewed data as median and [minimum – maximum]). Number of stimuli points within atrophy area (defined as RPEL) and junctional zone defined as EZL for each run on each device. Observations and descriptive statistics of drusen and HRF volume for values above 0.

### Mixed Effect Models

Tables 2 and 3 summarize results from univariate and multivariable mixed effect models with the smallest BIC (marginal *R*^2^_GLMM_ *=* 0.402), respectively. PWS was lower in areas with EZL, without RPEL compared with areas showing regular EZ integrity with a mean difference between −2 to −4 dB (95% confidence interval [CI] = estimate −3 dB). The strongest PWS reduction, between −9 dB and −11 dB (95% CI = estimate −10 dB), was observed in areas with RPEL and EZL compared with areas with intact EZ integrity and no RPEL.

**Table 2.** Univariate Model

Biomarker	Estimate	LL	UL	*P* Value	*n* Points
Drusen volume, nL	−2.08 dB	−2.92	−1.23	<0.0001	3557
HRF volume, nL	−23.30 dB	−27.86	−18.75	<0.0001	3557
EZT, µm	0.34 dB	0.32	0.36	<0.0001	3557
EZL area, µm^2^	−0.00062 dB	−0.00065	−0.00059	<0.0001	3557
RPEL area, µm^2^	−0.00083 dB	−0.00087	−0.00080	<0.0001	3557
Age	−0.067 dB	−0.33	0.20	0.60	3600
Eccentricity	0.562 dB	0.49	0.63	<0.0001	3600

dB, decibel; EZ, ellipsoid zone; EZT, ellipsoid zone thickness; HRF, hyper-reflective foci; *n* points, number of stimuli points; PWS, pointwise sensitivity; RPE, retinal pigment epithelium.

Effect of independent variables on PWS.

**Table 3.** Final Model

Biomarker	Estimate	LL	UL	*P* Value
MAIA vs. MP3	−3.36 dB	−3.69	−3.04	<0.0001
EZL, no RPE loss vs. no loss	−2.81 dB	−3.72	−1.91	<0.0001
EZL + RPEL vs. no loss	−10.03 dB	−10.96	−9.11	<0.0001
Eccentricity, R degrees	0.15 dB	0.083	0.22	<0.0001
EZL : R degrees	−0.31 dB	−0.50	−0.12	0.0011
EZL + RPEL : R degrees	−0.37 dB	−0.59	−0.15	0.0011

dB, decibel; EZL, ellipsoid zone loss; PWS, pointwise sensitivity; RPEL, retinal pigment epithelium loss.

Multivariable mixed model with the effect of EZL, RPEL, device and eccentricity on PWS. Only EZL was included due to strong multicollinearity with EZ thickness (*r* = 0.7). *Effect of eccentricity analyzed for category “no EZ/RPE loss.”

**Figure 2.**
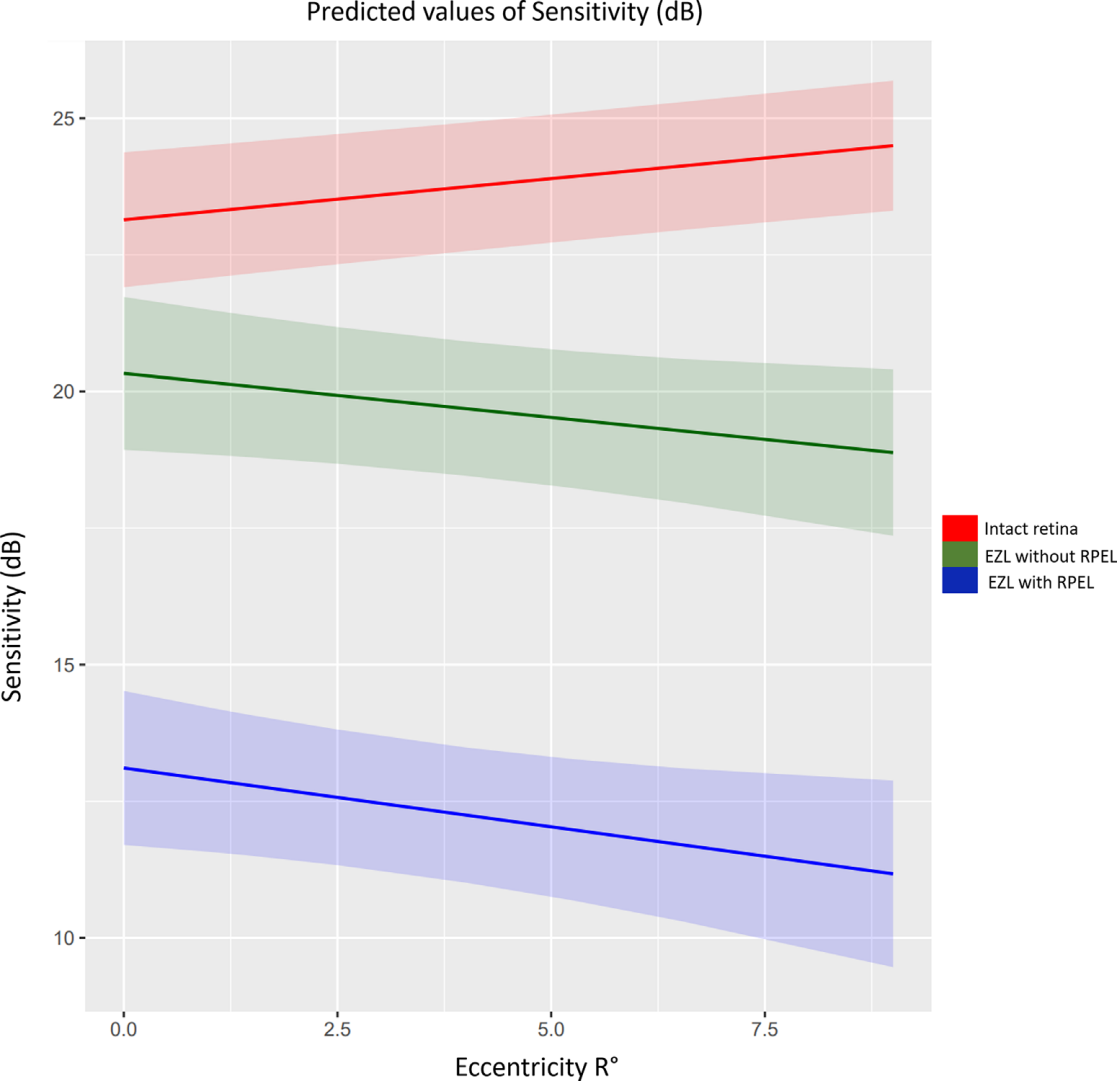
**Relationship between OCT biomarkers and function.** The *green line* represents the estimates and 95% CI of the effect of EZL area on PWS with increasing retinal eccentricity, whereas the *blue line* displays the effect and 95% CI of EZL and RPEL area on PWS with increasing retinal eccentricity. The *red line* corresponds to estimates with 95% CI within intact retina with increasing eccentricity. PWS is lower in areas with only EZL, no RPEL (*green line*) compared with healthy areas with intact EZ (*red line*) and in areas with EZL and RPEL (*blue line*) compared to EZL (*green line*) and healthy areas with intact EZ (*red line*). EZL = ellipsoid zone loss; RPEL = retinal pigment epithelium loss; PWS = pointwise sensitivity; CI = confidence interval.

Table 4 demonstrates changes in the estimates with a stronger reduction in PWS with increasing eccentricity (R degrees) of the respective stimuli. This relationship is graphically displayed in Figure 2. A strong Spearman correlation coefficient was found between RPEL and EZL (*r_s_* = 0.7), between EZ thickness and the EZL area (*r_s_* = −0.68) and the RPEL area (*r_s_* = 0.52). Due to strong multicollinearity between EZ thickness and EZL, only EZL was included in multivariable model. Still, univariate analyses revealed a significant reduction in PWS when EZ thickness decreased (0.34 dB/µm, 0.32–0.36, *P* < 0.0001).

**Table 4.** Final Model With Effect of Eccentricity.

R Degrees	Variable	Estimate	LL	UL	*P* Value
0 degrees	EZL vs. no loss	−2.81 dB	−3.72	−1.91	<0.0001
	EZL + RPEL vs. no loss	−10.03 dB	−10.96	−9.11	<0.0001
1.5 degrees	EZL vs. no loss	−3.28 dB	−3.97	−2.59	<0.0001
	EZL + RPEL vs. no loss	−10.58 dB	−11.25	−9.91	<0.0001
2.5 degrees	EZL vs. no loss	−3.59 dB	−4.16	−3.02	<0.0001
	EZL + RPEL vs. no loss	−10.95 dB	−11.50	−10.40	<0.0001
5.2 degrees	EZL vs. no loss	−4.43 dB	−4.96	−3.90	<0.0001
	EZL + RPEL vs. no loss	−11.94 dB	−12.56	−11.32	<0.0001

dB, decibel; EZL, ellipsoid zone loss; RPEL, retinal pigment epithelium loss.

Estimates derived from multivariable mixed effect model at different eccentricity values.

### Scotomatous Points

Table 5 demonstrates descriptive statistics of the probability of PWS of 0 dB, 5 dB, 10 dB, 15 dB, and 20 dB to be located within the area without atrophy, only the EZL area or the EZL and RPEL areas, as quantified by DL over all 3557 included measurements. There were 30.1% of stimuli points in the areas with EZL and RPEL had PWS of 0 dB, 37.4% had PWS of ≤ 5 and 49.5% had PWS of ≤ 10. Implementation of higher thresholds of 15 dB and 20 dB showed that 67.9% of PWS with atrophy, defined as EZL and RPEL was below 15 dB and 84.7% of PWS with atrophy was within areas below 20 dB.

**Table 5.** Number of Scotomatous Points.

PWS	No Atrophy	EZL No RPEL	EZL RPEL
=0	0.3%	3.4%	30.1%
≤5	0.9%	6.5%	37.4%
≤10	2.0%	14.4%	49.5%
≤15	7.9%	34.6%	67.9%
≤20	22.8%	61.0%	84.7%
Total	68.9%	15.7%	15.4%
	2452/3557	557/3557	548/3557

EZL, ellipsoid zone loss; PWS, pointwise sensitivity; RPEL, retinal pigment epithelium loss.

Percentage of stimuli points with PWS at different cut-off values within categories of no EZL and RPEL (no atrophy), EZL without RPEL and both, EZL and RPEL. The last line corresponds to a contingency table of area with no atrophy, with EZL without RPEL and EZL with RPEL independent of PWS.

## Discussion

Our findings demonstrate a consistent pointwise structural correspondence between DL-quantified key OCT biomarkers of GA and function in MP with a pivotal personalized assessment of functional changes in the perilesional area. OCT-derived areas of RPEL and EZL were topographically associated with significantly reduced retinal sensitivity with important insights into GA pathophysiology, accounting for age, distance from fovea, HRF, and drusen volume. This result was consistent in the final multivariate model with significant differences between MAIA and MP-3, as well as for each device separately.

Meleth et al. proposed MP as a functional end point in GA trials as early as 2011.^15^ Since then, various groups used different metrics to address the need for reliable and objective functional endpoints in GA. A significantly greater reduction in mean retinal sensitivity in eyes with GA compared with controls suggests a general functional impairment in eyes with GA.^13^ However, although longitudinal studies demonstrate an overall mean retinal sensitivity decrease in patients with GA and proven sensitivity changes to be a strong biomarker to predict conversion from early to late non-exudative AMD, only a weak correlation was found with GA lesion progression.^8^^,^^30^ Our study defines functional reduction in prespecified OCT areas, while mean reduction of −9 to 11 dB in areas with RPEL are comparable to a previously published mean decrease of 9 dB in GA areas delineated on fundus autofluorescence (FAF).^9^ Concomitantly, early work using manual grading of spectral-domain OCT (SD-OCT) images found that RPE thinning was a significant predictor of absolute scotoma in MP-1, which was demonstrated in our dataset based on automated, that is, precision-based RPEL quantification.^31^ Chang et al. proposed sensitivity changes in the perilesional area as a more sensitive metric to mean sensitivity change. This region was previously defined in general terms as a universal circumference of 250 or 500 µm outside the atrophy border and named junctional zone and/or perilesional area.^32^ Vogl et al. demonstrated that EZL surrounds GA lesions on OCT,^33^ and showed a large variability in the condition of the junctional zone with implications for disease monitoring and progression.^34^^,^^35^ AI-based analysis of structural changes at the EZ level in the phase III approval studies for C3-complement inhibition demonstrated a strong impact on EZ maintenance in the surrounding of the GA lesion which was not identifiable by standard FAF evaluation.^34^ The therapeutic effect on the areas of EZ loss outside of the clinical lesion was even superior to the inhibition of RPE loss progression. The knowledge that EZ loss is consistent with PR degeneration and has a defined association with functional loss suggests AI-based monitoring for GA management in clinical practice. The US Food and Drug Administration (FDA) has already recognized EZ attenuation as a valid biomarker in GA clinical trials.^36^ Takahashi et al. found a mean difference of 4 dB in atrophy surrounding areas with PR damage compared to no damage, respectively.^9^ Our findings in individually defined perilesional/atrophy surrounding areas with a PWS decrease of −3 dB compared with the healthy areas are in line with this finding. Importantly, in areas with EZ loss, there is a significant functional decrease, not functional loss, as OCT imaging shows degeneration to an extent where there is no EZ present, but other parts of the PR might still be preserved. Strong multicollinearity between EZ thickness and EZL (*r_s_* = −0.7) was proven as expected. To avoid model overloading, only the most pathognomonic biomarkers of GA as defined by international OCT consensus,^37^^,^^38^ EZL and RPEL were considered in the multivariable mixed model. Note that EZ thickness measurements were available in 90% of stimuli points, which strengthens this result. Post hoc subset analysis of both devices separately highlighted the consistency of this result with a significant decline of PWS in predefined OCT areas. Function decreased significantly with higher eccentricity for MP3, but not significantly for MAIA (Supplementary Table S2). The distribution of rods and cones along the macular region plays into this finding, as photopic testing excites cone signals which are located at the central foveal region and mesopic testing excites, both cone and rod signals, distributed in parafoveal regions. This highlights how the effect of eccentricity is influenced by the chosen background luminance in the chosen MP device.

**Figure 3.**
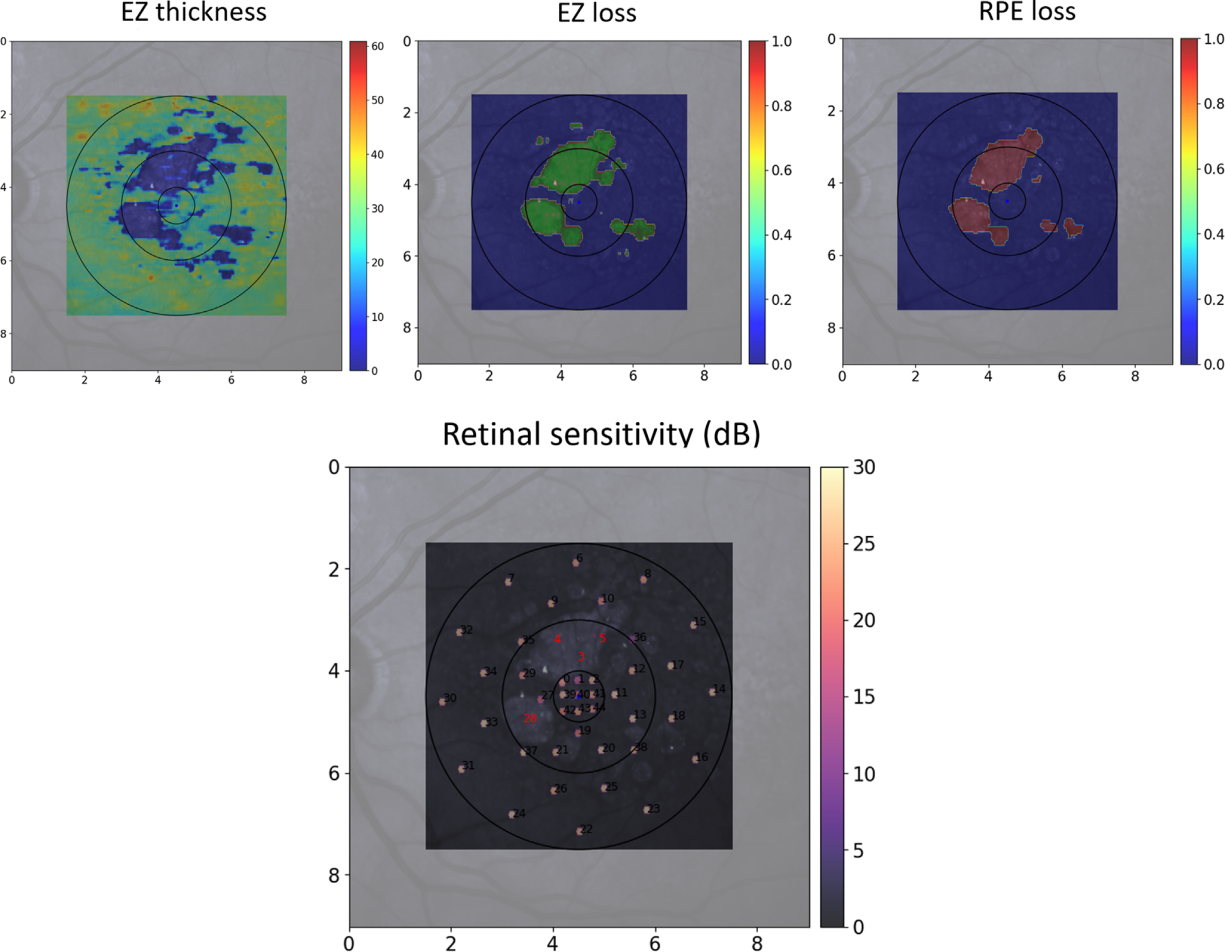
**En face area of EZ thickness, EZ loss,** **and RPE loss.** The *upper row* displays en face heat maps of EZ thickness with the color scale corresponding to the respective EZ thickness change in µm (*left*) and area of EZL (*green, middle*) and RPEL (*red, right*) from OCT volumes. The *lower row* represents the superimposed customized 45-point MP grid within the Spectralis NIR. The *red marked numbers* represent MP points with sensitivity values below 10 dB and lie within the area with EZL and PREL on en face NIR. Numbers in the *lower row* correspond to stimuli point numbers, not PWS values. OCT = optical coherence tomography; RPE = retinal pigment epithelium; EZ = ellipsoid zone; MP = microperimetry; NIR = near-infrared reflectance.

For functional assessment of GA, another proposed method was the analysis of scotomas points, which previously showed a strong correlation with GA growth.^8^^,^^15^ Notably, in our cohort, only 5% of stimuli points had absolute scotomas of 0 dB and only 11% of points had PWS below 10 dB. Figure 3 shows an example of PWS below 10 dB (marked as red numbers) located within an area, defined as the EZL and RPEL areas. We examined higher thresholds within RPEL areas and found evidence for residual light sensitivity within atrophy areas, as previously published (see Table 5).^39^ The use of high-density grids was proposed as an alternative method to catch functional decline in GA in recent literature.^14^ Targeted areas of interest with incomplete outer retinal atrophy had significantly reduced sensitivity compared with fixed MP grids. Both methods, absolute scotomas and lesion-targeted grids, need further validation in longitudinal studies to establish the clinical feasibility during follow-up.^14^^,^^15^ Still, our method also needs to be validated during follow-up. Personalized MP grids based on real-time EZL and RPEL quantification in OCT volumes might further optimize the output of targeted structure/function assessment.

Interestingly, univariate analyses revealed a significant negative effect of drusen and HRF volume on PWS, which is in line with current literature in intermediate AMD.^20^^,^^23^ Note that median HRF volume was 0.10 to 0.11 nL, which would change the estimate to −0.23 dB/0.01 nL. The strong functional change over HRF can be explained by the previously described HRF location in areas with outer retinal alterations, such as RPE migration and loss (Supplementary Fig. S1). To understand the cycle of drusen regression and AMD progression with respect to functional outcomes, longitudinal studies seem better suited, as well as structure-function correlation in eyes with intermediate AMD.^23^

The major strength of our analyses is the prospective design and the innovative method combining semi-automated image registration algorithms and automated OCT biomarker quantification, that is, MP based on clearly delineated areas with defined levels of structural alteration, while also accounting for other high-risk biomarkers of drusen and HRF. In addition, we established the structure-function relationship in two MP devices and included the impact of foveal eccentricity. Notably, significant differences between MP-3 and MAIA are likely due to different standard settings in both devices, photopic background for MP-3 and mesopic background for MAIA, respectively. Our cohort encompasses patients with and without foveal sparing to reflect the broad representation of GA. We interpret the applicability and consistency of our results to two different MP devices as a strength of our analyses. The study has several limitations that should be considered. First, despite pivotal findings, the data analyses can only be viewed as exploratory in a small cohort of 20 eyes, whereas acquisition in MP with a 45-point grid and patient compliance remain limiting factors. Notably, we analyzed the data on a stimulus point basis to address this limitation and included 3600 PWS values with 90% with EZ thickness measurements. Second, the lack of longitudinal data to further validate our method during follow-up should be addressed in bigger longitudinal datasets. Third, although the registration process was performed in a semi-automated manner with certified human expertise, minimal deviations of the location of stimuli points on the respective MP device and the analyzed pixel on the OCT during the registration process are inevitable. Fourth, due to a predefined grid position, only a limited amount of PWS was located within the atrophy area.

In GA, an objective and quantified assessment of function is essential for understanding the pathomechanisms of progression, for optimal clinical management and to develop treatment which leads to functional benefit. MP is a valid tool to provide such structure/function associations, but is highly dependent on the topographic condition of the tested area. Moreover, structural changes are mostly subclinical in GA such as EZ thinning and loss. Topographic pointwise correlation between DL-defined GA biomarkers and MP shows a high potential for personalized definition of the perilesional changes to understand the pathophysiology in GA and target regions of interest. These fill the knowledge gap between OCT morphology and functional testing for the evaluation of individualized disease activity and novel interventional targets in patients with GA.

## Supplementary Material

Supplement 1
