# Supplementary material for: Structure-Function Correlation of Deep-Learning Quantified Ellipsoid Zone and Retinal Pigment Epithelium Loss and Microperimetry in Geographic Atrophy
Source: Invest Ophthalmol Vis Sci. 2025 Mar 11;66(3):26. doi: 10.1167/iovs.66.3.26 (PMC11918028; doi:10.1167/iovs.66.3.26)
Supplement: Supplement 1 [file iovs-66-3-26_s001.pdf]

Supplementary material

	Overall		
	Fovea	Parafovea	Perifovea
**Drusen volume (nl)**	7.81 ± 12.19	54.45 ± 43.35	95.07 ± 81.51
**HRF volume (nl)**	1.27± 1.16	9.76 ± 4.73	17.07 ± 8.9
**EZ loss area (mm^2)**	0.27 ± 0. 25	2.48 ± 1. 4	1.98 ± 2.25
**RPE loss area (mm^2)**	0.15 ± 0.21	1.26 ± 0.86	0.83 ± 1.35
		**Pointwise**	
	MP stimuli Fovea	MP stimuli Parafovea	MP stimuli Perifovea
**Number of MP stimulus points**	n =720	n = 1120	n = 1397
**Drusen volume (nl)**	0.23 ± 0. 39	0.20 ± 0.29	0.18 ± 0.32
**HRF volume (nl)**	0.15 ± 0.12	0.14 ± 0.09	0.13 ± 0.08
**EZ loss presence**	321/720 45%	533/1120 48%	139/1397 10%
**RPE loss presence**	174/720 24%	300/1120 27%	37/1397 3%

Supplementary Table 1: Descriptive statistics on overall RPE loss and therefore atrophy size, EZL, drusen and HRF volume throughout the whole OCT volume scans in the foveal region, parafoveal region and perifoveal region (upper part of table). Descriptive statistics on pointwise HRF and drusen volume for MP stimuli located within central 1 mm, central 3 mm and central 6 ring, including the number of stimuli located within atrophy area (RPEL) and EZL area. 4/45 MP stimuli points are located at transition zones between the 1,3- and 6-mm rings and were not included to this table (lower part of table). EZL = ellipsoid zone loss, RPEL = retinal pigment epithelium loss, HRF = hyperreflective foci, MP = microperimetry

MP 3				
Variable	Estimate	LL	UL	p value
EZL	-3.108	-4.387	-1.829	**<0.0001**
RPEL	-12.729	-14.029	-11.429	**<0.0001**
R°	0.199	0.107	0.290	**<0.0001**
EZL*R°	-0.276	-0.536	-0.017	**0.0367**
RPEL*R°	-0.088	-0.391	0.214	0.5666
MAIA				
EZL	-2.630	-3.885	-1.374	**<0.0001**
RPEL	-7.853	-9.138	-6.568	**<0.0001**
R°	0.094	-0.003	0.191	0.0575
EZL*R°	-0.350	-0.614	-0.086	**0.0095**
RPEL*R°	-0.566	-0.879	-0.253	**0.0004**

Supplementary Table 2: Subset analysis of mixed effect models for each device separately. MAIA was performed with a mesopic background setting and MP-3 was performed with a photopic background setting. EZ = ellipsoid zone loss, RPEL = retinal pigment epithelium loss, R° = retinal eccentricity

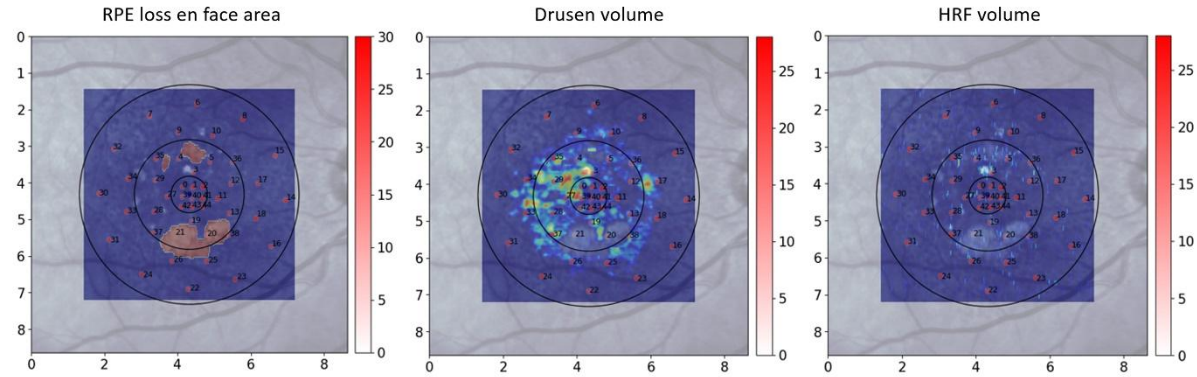

Supplementary Figure 1: Topographic relation in en face heatmap of drusen volume, HRF volume and cRORA location defined as RPEL quantified on OCT. HRF = hyperreflective foci, cRORA = complete outer retinal atrophy, RPEL = retinal pigment epithelium loss, OCT = optical coherence tomography
